# Circulating lipocalin-2 is associated with systemic inflammation and cachexia-related features in patients with gastrointestinal cancer

**DOI:** 10.3389/fnut.2026.1937525

**Published:** 2026-10-19

**Authors:** Federica Tambaro, Giovanni Imbimbo, Dario Coletti, Roberta De Tullio, Elena Belloni, Giuseppe Nigri, Paola Costelli, Maurizio Muscaritoli, Alessio Molfino

**Affiliations:** 1 Department of Translational and Precision Medicine, Sapienza University of Rome, Rome, Italy; 2 DAHFMO Unit of Histology and Medical Embryology, and Interuniversity Institute of Myology, Sapienza University of Rome, Rome, Italy; 3 Department of Experimental Medicine (DIMES), Section of Biochemistry, University of Genova, Genova, Italy; 4 Department of Medical-Surgical Sciences and Translational Medicine, Sapienza University of Rome, Rome, Italy; 5 Department of Clinical and Biological Sciences, University of Turin, Turin, Italy; 6 Department of Interdisciplinary Studies on WellBeing, Health and Environmental Sustainability (BeSSA), Sapienza University of Rome, Rome, Italy

**Keywords:** anorexia, cachexia, cytokines, gastrointestinal cancer (GI), lipolocalin-2, muscle wasting, systemic inflamamtion

## Abstract

**Background:**

Cancer-associated anorexia (AN) and cachexia are frequent and debilitating complications of cancer, driven by systemic inflammation and metabolic dysregulation. Lipocalin 2 (LCN2) has been implicated in immune-metabolic integration and central appetite control, however data in cancer patients are limited. This study aimed to assess circulating LCN2 in patients with gastrointestinal cancers (GI-CP) and to evaluate its association with AN, levels of systemic inflammatory cytokines (IL-6, −8, −10, −15, −18, and TNF-α), body weight change, and level of muscularity.

**Methods:**

Twenty-eight GI-CP naïve to treatment and 10 controls were enrolled. AN was diagnosed in 50% of GI-CP by the Functional Assessment of Anorexia/Cachexia Therapy (FAACT) score ( ≤ 30). Serum LCN2 concentrations were measured by ELISA, and cytokines were quantified using xMAP assay. Clinical, nutritional, and body composition parameters assessed by CT-scan were collected, and association(s) between LCN2 and inflammatory markers were analyzed.

**Results:**

In GI-CP, predominantly represented by colorectal cancer, LCN2, IL-6, IL-8, IL-15, and TNF-α were higher than controls (*p* < 0.05). In GI-CP, LCN2 showed positive correlations with IL-6 (rho = 0.862; *p* < 0.001) and IL-8 (rho = 0.789; *p* < 0.001). LCN2 levels were higher in GI-CP with AN vs. control (*p* = 0.003), but not between anorexic vs. non-anorexic GI-CP, and in GI-CP with body weight loss compared to those with weight-stable (*p* = 0.031) and vs. controls (*p* < 0.001). LCN2 was higher in GI-CP with AN and body weight loss compared to those without AN and body weight stable (*p* = 0.046) and in GI-CP with low muscle mass and body weight loss compared with those with body weight stable and high muscularity (*p* = 0.028).

**Conclusion:**

Overall, these findings suggest that LCN2 may reflect the combined burden of inflammation and cachexia-related features in GI cancer.

## Introduction

Cancer-induced anorexia and cachexia are tightly intertwined multifactorial metabolic syndromes characterized by reduced food intake, involuntary weight loss, systemic inflammation, and skeletal muscle and adipose tissue wasting, affecting up to 80% of patients with advanced gastrointestinal malignancies, including pancreatic and gastric cancers (1). These conditions severely impair quality of life, reduce tolerance to anticancer therapies, and predict poor clinical outcomes, especially increased mortality (2). Notably, anorexia often precedes overt weight loss and muscle depletion, suggesting that early alterations in appetite regulation may contribute to the subsequent development of hypophagia and cachexia (3). Despite their clinical relevance and impact, the molecular mechanisms linking systemic inflammation, nutritional impairment, and tissue wasting remain not completely understood, and reliable circulating biomarkers for early identification and risk stratification are still lacking.

Lipocalin-2 (LCN2), also known as neutrophil gelatinase-associated lipocalin (NGAL), is an acute-phase protein involved in innate immunity, iron homeostasis, and inflammatory responses (4). Beyond its established role in infections and inflammation, emerging evidence suggests that LCN2 acts as a neuroendocrine hormone that binds the melanocortin-4 receptor (MC4R) in the hypothalamic paraventricular nucleus to suppress appetite, regulate energy balance, and modulate muscle metabolism (5–7). Elevated circulating LCN2 levels have been reported in several inflammatory and catabolic conditions, including cancer, where they often correlate with disease progression and poor patient outcomes (8, 9). Preclinical studies further suggest that LCN2 contributes to anorexia and to skeletal muscle and adipose tissue wasting, positioning it as a potential upstream regulator of cancer-associated metabolic derangements (7, 10).

Systemic inflammation is a central driver of cancer cachexia (11), and the interaction between LCN2 and pro-inflammatory cytokines such as IL-6 and TNF-α may represent a critical axis contributing to nutritional deterioration and muscle wasting in cancer patients. Within this context, LCN2 may act as an integrative signal linking inflammatory pathways to central and peripheral mechanisms of nutritional decline.

By this study, we aimed to assess the circulating levels of LCN-2 in patients with gastrointestinal cancers compared with non-cancer controls and to examine their association with inflammatory/anti-inflammatory cytokines, including IL-6, IL-8, IL-10, IL-15, IL-18 and TNF-α. We also evaluated the association between circulating LCN2 and key clinical and nutritional features of cancer cachexia, with particular attention to anorexia, involuntary weight loss, and skeletal muscle mass.

## Materials and methods

### Study design and patient’s enrollment

This single-center, observational, cross-sectional study considered consecutive patients diagnosed with gastrointestinal cancers (gastric, pancreatic, and colorectal) at the Department of Medical-Surgical Sciences and Translational Medicine at Sapienza University of Rome, Italy. All experimental procedures were conducted at the Department of Translational and Precision Medicine within the same Institution.

Patients were enrolled upon diagnosis, prior to any surgical or oncological treatment, among those scheduled for surgical tumor resection. Inclusion criteria included: age ≥18 years, histologically confirmed gastrointestinal cancer diagnosed within the previous 4 weeks, and no prior exposure to anticancer, corticosteroid, or anti-inflammatory treatments. Exclusion criteria included: newly diagnosed patients with advanced-stage cancer for whom surgical resection was not indicated, acute or chronic conditions known to affect nutritional status or systemic inflammation, including active infections, hepatic or renal failure, heart failure, autoimmune diseases, severe malabsorption syndromes, intestinal obstruction, dysphagia, or other inflammatory conditions.

The control group consisted of individuals undergoing elective surgery for non-malignant conditions, with no history of cancer or inflammatory diseases.

The study was conducted in accordance with the Declaration of Helsinki and received approval from the Local Ethics Committee of Sapienza University, Azienda Sant’Andrea Hospital, Rome, Italy (protocol number 167_2017).

All participants provided written informed consent prior to study participation.

### Clinical and nutritional assessment

At enrollment, comprehensive clinical, anthropometric and nutritional assessments were performed for all participants, including demographic data, medical history, comorbidities, tumor staging, and histological classification.

Body weight (kg) and height (m) were measured during the study visit, and body mass index (BMI, kg/m^2^) was calculated. The presence or absence of involuntary body-weight loss during the previous 6 months was recorded for all participants.

### Assessment of anorexia

Anorexia was assessed using the Italian version of the Functional Assessment of Anorexia/Cachexia Therapy (FAACT) questionnaire. The FAACT comprises 12 items scored on a 5-point Likert scale (0 = not at all to 4 = very much), yielding a total score ranging from 0 to 48, as previously described (12). Patients were classified as anorexic (FAACT ≤ 30) or non-anorexic (FAACT > 30), according to previously validated cut-offs (12). The questionnaire was administered during the first oncology visit, with assistance from medical personnel.

### Body composition assessment

Skeletal muscle mass was assessed using abdominal computed tomography (CT) scans performed for routine clinical purposes. Axial slices at the level of the third lumbar vertebra (L3) were analyzed to quantify total skeletal muscle area (SMA), including *psoas*, paraspinal, and abdominal wall muscles. Image analysis was performed using OsiriX Lite DICOM Viewer (version 11.0.3). Skeletal muscle tissue was identified using a predefined attenuation range of −29 to +150 Hounsfield units. The skeletal muscle index (L3-SMI, cm^2^/m^2^) was calculated by normalizing SMA to height squared.

Low muscle mass (LMM) was defined as median value of L3-SMI with internal sex-specific cut-off set as 47.89 cm^2^/m^2^ for males and 39.14 cm^2^/m^2^ for females.

### Blood sample collection and processing

Fasting peripheral blood samples were collected into additive-free Vacutainer® Serum tubes and incubated at room temperature for at least 30 min to induce clotting. Serum was separated by centrifugation at 1,500 × g for 10 min at 4 °C, aliquoted, and stored at −80 °C until analysis.

### Serum lipocalin-2 measurement

Serum concentrations of LCN2 (NGAL) were quantified using a commercially available enzyme-linked immunosorbent assay (ELISA) kit (cat. #A101877, Antibodies.com, Cambridge, UK), according to the manufacturer’s instructions. Serum samples were diluted 1:20 before analysis and were analyzed in duplicate. Absorbance was measured at 450 nm using the Multiskan Sky spectrophotometer (Thermo Fisher Scientific, Waltham, MA, USA). Concentrations were calculated by interpolation from the standard curve and corrected for the corresponding dilution factor. The lower limit of detection of the assay was 79 pg./mL. Final circulating LCN2 concentrations were expressed in ng/mL. According to the manufacturer’s technical specifications, the intra-assay CV is <10% and the inter-assay CV is <12%.

### Serum cytokines multiplex assay

Serum concentration of IL-6, IL-8, IL-10, IL-15, IL-18 and TNF-α were quantified using the custom human premixed multiplex magnetic Luminex® assay (cat. #LXSAHM-06, Bio-Techne, San Francisco, CA, USA). Cytokine profiling was performed according to the manufacturer’s instructions. Briefly, serum samples were diluted (1:1 vol/vol) with assay buffer. Samples were incubated with microparticle cocktail in a 96-well plate (overnight at 4 °C). Biotin-antibody was then added (1 h at room temperature), followed by Streptavidin-PE solution (30 min at room temperature). All samples were analyzed in duplicate on a Luminex® xMAP® technology (Luminex Corp., Austin, TX, USA). The intra-assay coefficient of variation (CV) limit was set at 20%, and all measurements fell within this range. Recovery rates for the assays ranged between 80% and 120%. An eight-point standard curve was generated for each cytokine using a five-parameter logistic (5-PL) regression model. Data acquisition was conducted using xPONENT software and Quantist Luminex® Data Analysis Software (Bio-Techne, San Francisco, CA, USA) was employed for the analysis. Cytokine concentrations were expressed in pg./mL.

### Statistical analyses

Data are presented as mean ± standard deviation (SD) or median with interquartile range (IQR) for normally or non-normally distributed variables, respectively.

Student’s *t*-test or one-way ANOVA was used for comparisons of normally distributed variables, while the Mann–Whitney U test or Kruskal–Wallis test was applied for non-normally distributed data.

Spearman’s correlation coefficient (rho) was used to assess associations between circulating LCN2 levels and cytokines, as well as clinical and nutritional parameters. Given their exploratory and hypothesis-generating nature, analyses examining the relationships between LCN2 and inflammatory cytokines, anorexia, body-weight change, muscularity, and combined clinical phenotypes were not formally adjusted for multiple comparison.

Finally, a multivariable logistic regression model was additionally performed with body-weight loss as the dependent variable and circulating LCN2, age, sex, and CRP as independent variables. Results are reported as odds ratios (ORs) with 95% confidence intervals (CIs). A two-tailed *p*-value < 0.05 was considered statistically significant. Statistical analyses were performed using IBM® SPSS Statistics version 30.0 (SPSS Inc., Chicago, IL, USA) and GraphPad Prism 10.2.3 (San Diego, California, USA).

## Results

### Patient’s characteristics

The study included 28 patients with gastrointestinal cancer and 10 subjects serving as controls. Among gastrointestinal cancer patients, 68% were male with a median age of 69.5 years (53.5; 78.75), whereas 50% of controls were male with median age of 48.0 years (39.0; 77.0). Demographic, anthropometric and clinical characteristics are summarized in Table 1.

**Table 1:** Clinical and demographic characteristics of the study population.

Parameter	Gastrointestinal cancer patients *n* = 28	Controls *n* = 10	*p*-value
Male, *n* (%)	19 (68)	5 (50)	
Age (years), median (IQR)	69.5 (53.5; 78.75)	48.0 (39.0; 77.0)	*p* = 0.065
Actual weight, kg	67.5 (61.5; 81.8)	70.0 (67.0; 70.0)	*p* = 0.971
BMI (kg/m^2^), mean ± SD	23.28 ± 3.25	24.23 ± 2.68	*p* = 0.295
Weight loss in previous 6 months, *n* (%)	16 (57)	/	
Blood parameters			
C-reactive protein, mg/dL	5.07 (2.57; 12.62)	1.65 (0.50; 3.40)	*p* = 0.156
Haemoglobin, g/dL	11.79 ± 1.90	12.57 ± 1.70	*p* = 0.080
Albumin, g/dL	3.45 ± 0.53	3.51 ± 0.13	*p* = 0.348
Creatinine, mg/dL	0.84 (0.70; 1.31)	0.73 (0.67; 0.86)	*p* = 0.244
eGFR, mL/min/1.73 m^2^	88.2 (61.8; 102.2)	102.8 (87.4; 107.8)	*p* = 0.052
Main comorbidities			
Diabetes mellitus, *n* (%)	5 (18%)	/	
Dyslipidemia, *n* (%)	6 (21%)	/	
Type of cancer			
Colorectal, *n* (%)	24 (86%)		
Gastric, *n* (%)	3 (11%)		
Pancreas, *n* (%)	1 (4%)		
Stage of the disease, *n* (%)			
I–II	16 (57%)		
III–IV	12 (43%)		
Anorexia according to FAACT score ≤ 30, *n* (%)	14 (50%)	/	

BMI, body mass index; eGFR, estimated glomerular filtration rate; FAACT, Functional Assessment of Anorexia/Cachexia Therapy; SD, standard deviation; IQR, interquartile range.

Among gastrointestinal cancer patients, 16 out of 28 (57%) reported involuntary weight loss in the previous 6 months. In the cancer cohort, tumor localization was predominantly colorectal (*n* = 24), followed by gastric (*n* = 3) and pancreatic cancer (*n* = 1). According to disease staging, 57% of patients were classified as stage I-II, while 43% were stage III-IV.

Based on the Functional Assessment of Anorexia/Cachexia Therapy (FAACT) score, anorexia (FAACT ≤ 30) was present in 50% of gastrointestinal cancer patients.

The demographic, anthropometric, and clinical characteristics of the subgroups (i.e., anorexic vs. non anorexic patients, body weight loss vs. body weight stable patients as well as patients with low muscle mass vs. high muscle mass) are reported in Supplementary Tables S1–S3.

### Circulating lipocalin 2 levels and inflammatory cytokine profile in gastrointestinal cancer and controls

As shown in Figure 1A, serum LCN2 levels were significantly higher in patients with gastrointestinal cancer compared with controls (*p* < 0.001) (Figure 1A). Serum LCN2 levels did not correlate with age in both cancer patients and controls (*r* = 0.115, *p* = 0.561 and *r* = −0.157, *p* = 0.666; respectively).

**Figure 1:**
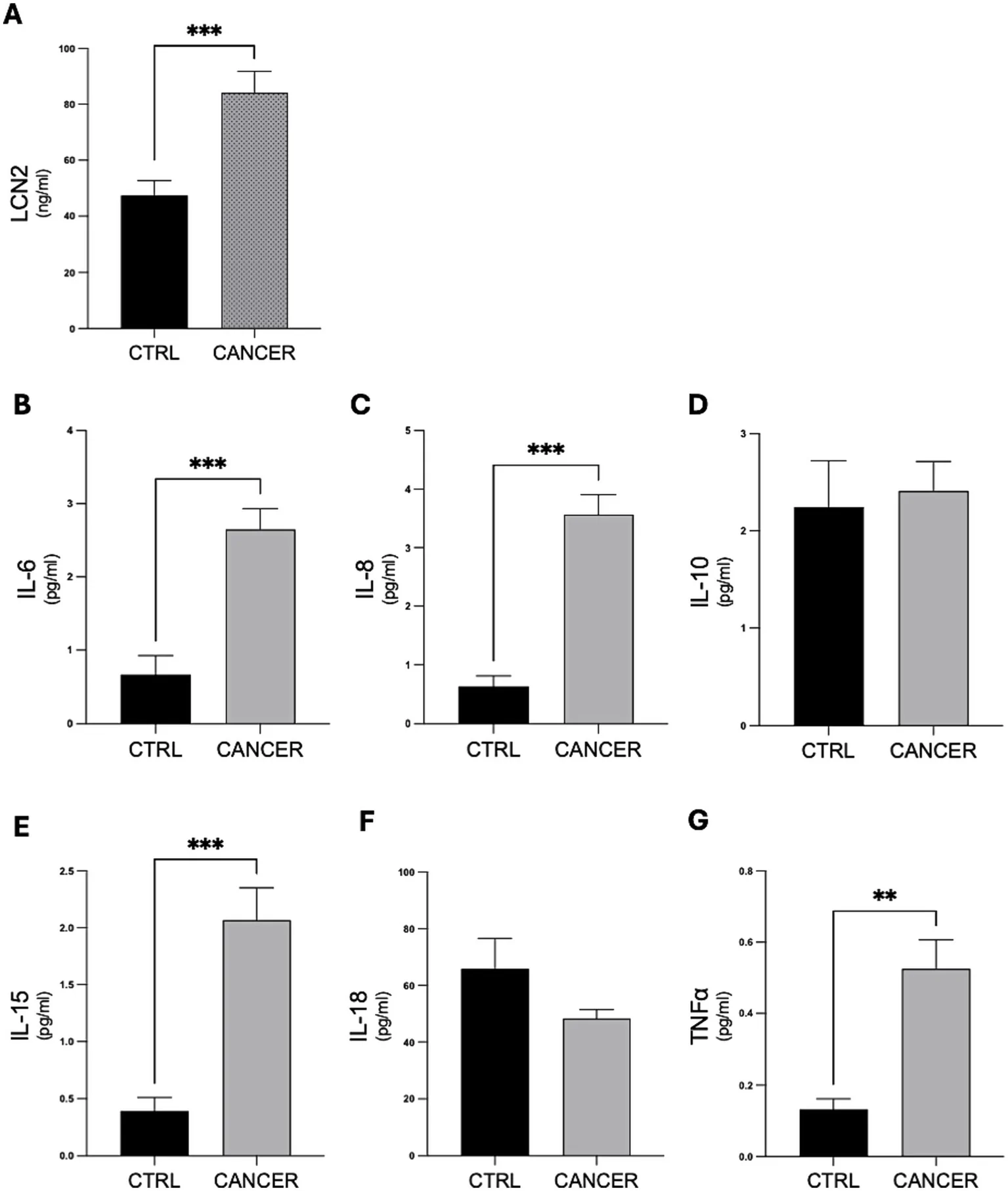
Differences in circulating levels of lipocalin 2 and cytokines in patients with gastrointestinal cancer and controls. **(A)** Circulating LCN2 concentrations were measured by ELISA assay. Data showed significantly higher LCN2 levels in patients with gastrointestinal cancer (*n* = 28) compared to controls (*n* = 10) (*p* < 0.001). **(B–G)** Cytokines concentrations were measured by xMAP assay in patients with gastrointestinal cancer (*n* = 28) compared to controls (*n* = 10). Data shown significantly higher IL-6 (*p* < 0.001) **(B)**, IL-8 (*p* < 0.001) **(C)**, IL-15 (*p* < 0.001) **(E)** and TNF-α (*p* = 0.002) **(G)** levels in patients with gastrointestinal cancer vs. controls. No significant differences were observed for IL-10 **(D)** and IL-18 **(F)**. Data are presented as mean ± SEM. *Student’s t-test* or Mann–Whitney test were applied for normally or non-normally distributed data, respectively. * *p* < 0.05, ** *p* < 0.01, *** *p* < 0.001. LCN2, lipocalin 2; IL-6, interleukin-6; IL-8, interleukin-8; IL-10, interleukin-10; IL-15, interleukin-15; IL-18, interleukin-18; TNF-α, tumor necrosis factor alpha.

Cancer patients exhibited a markedly altered inflammatory cytokine profile compared to controls, characterized by significantly increased circulating levels of IL-6, IL-8, IL-15, and TNF-α compared with controls (all *p* < 0.05), whereas no significant differences were observed for IL-10 and IL-18 (Figures 1B–G).

Spearman’s rank analysis of the cancer cohort demonstrated that LCN2 levels were significantly positively correlated with IL-6, IL-8, IL-15 and TNF-α, and inversely correlated with IL-10 and IL-18 (*p* < 0.01) (Figure 2).

**Figure 2:**
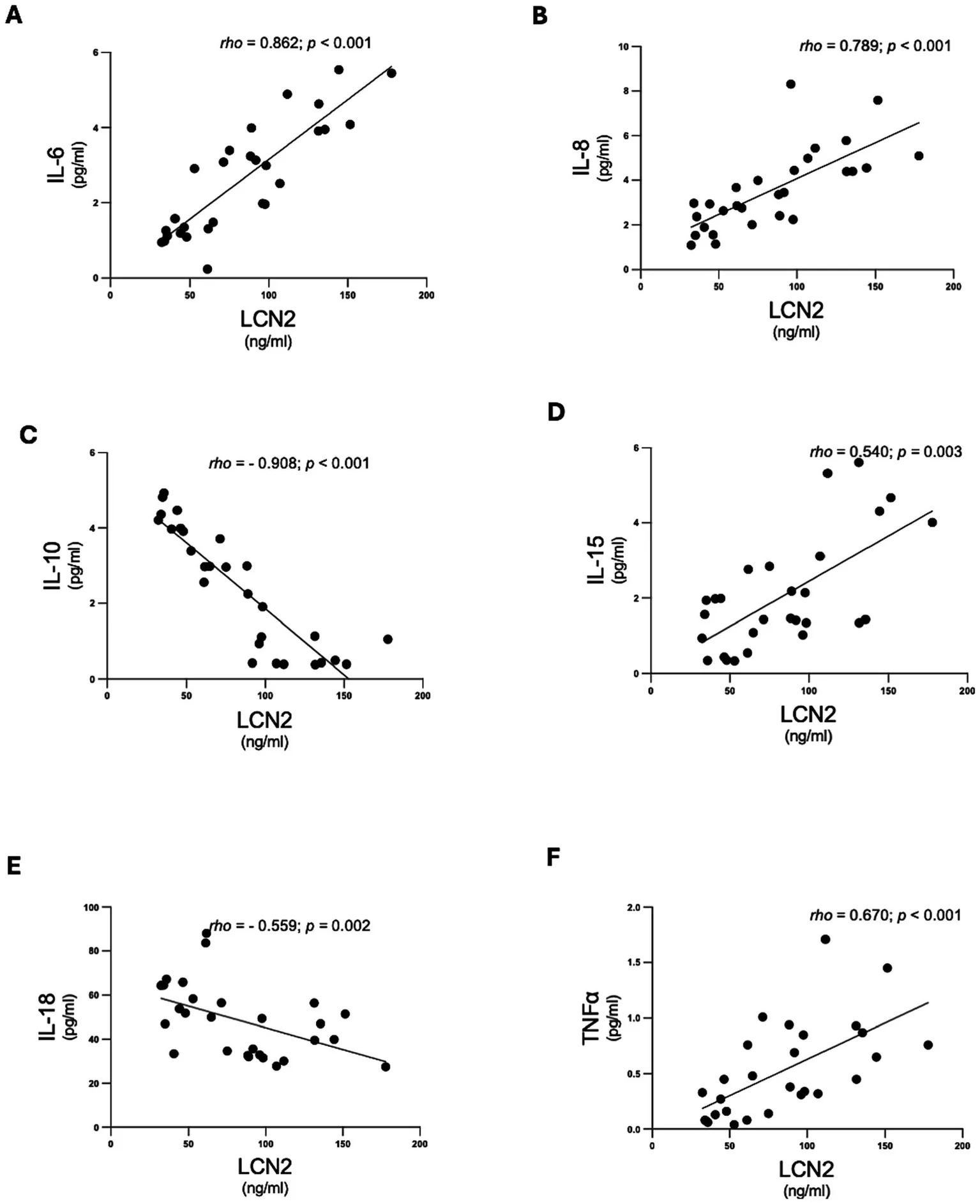
Spearman’s correlation analysis between circulating LCN2 and cytokines in patients with gastrointestinal cancer. Spearman’s correlation analyses were performed between circulating LCN2 levels and IL-6 (rho = 0.862, *p* < 0.001) **(A)**, IL-8 (rho = 0.789, *p* < 0.001) **(B)**, IL-10 (rho = −0.908, *p* < 0.001) **(C)**, IL-15 (rho = 0.540, *p* = 0.003) **(D)**, IL-18 (rho = −0.559, *p* = 0.002) **(E)**, and TNF-α (rho = 0.670, *p* < 0.001) **(F)**. LCN2, lipocalin 2; IL-6, interleukin-6; IL-8, interleukin-8; IL-10, interleukin-10; IL-15, interleukin-15; IL-18, interleukin-18; TNF-α, tumor necrosis factor alpha.

### Circulating levels of lipocalin 2 in relation with anorexia, body weight change, and inflammatory cytokines

We found significant increased LCN2 levels in anorexic cancer patients vs. controls (*p* = 0.003) (Figure 3A). In parallel, subgroup stratification by anorexia status yielded significant correlations between LCN2 and inflammatory cytokines (Table 2 - left side).

**Figure 3:**
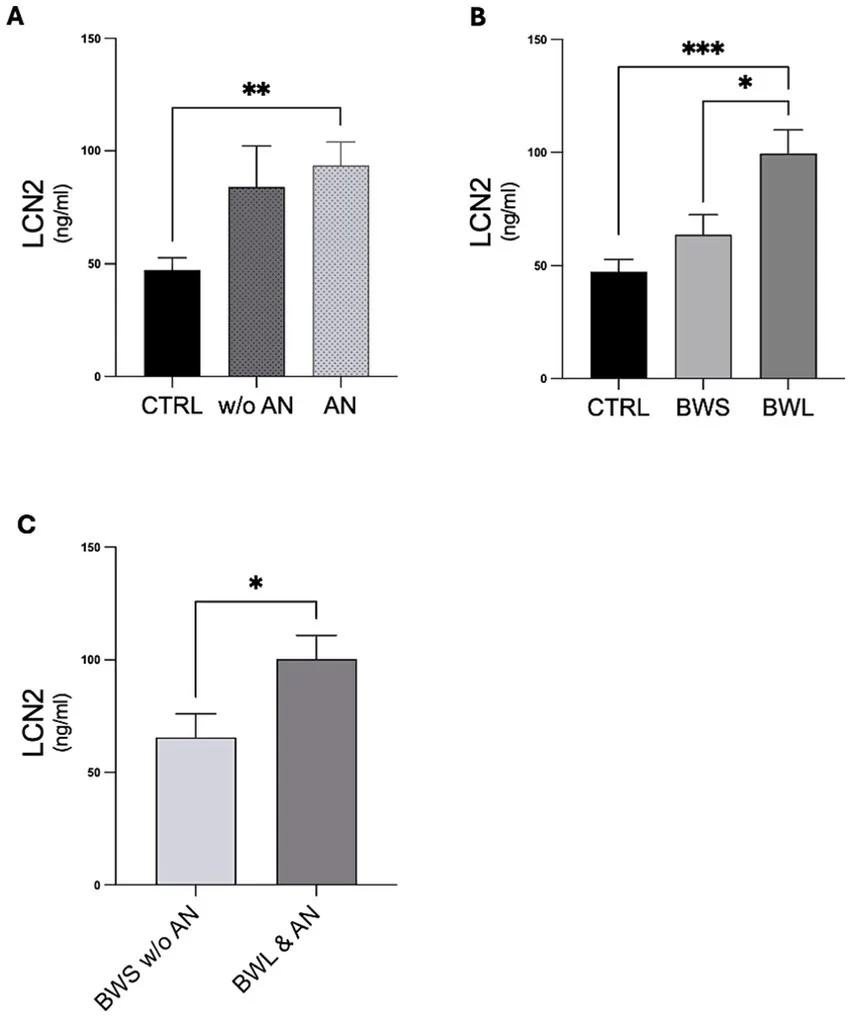
Differences in circulating levels of lipocalin 2 in relation to clinical and anorexia status. **(A)** LCN2 levels were analyzed according to anorexia (AN) status based on the Functional Assessment of Anorexia/Cachexia Therapy (FAACT) score. Groups included: patients without AN (w/o AN) (FAACT>30; *n* = 14), presence of AN (FAACT≤30; *n* = 14) vs. controls (*n* = 10). Data shown significantly higher LCN2 levels in AN compared to controls (*p* = 0.003). **(B)** LCN2 levels were analyzed according to patients self-reported 6-month weight changes prior to enrollment and controls. Significantly higher LCN2 levels were observed in patients with body weight loss (BWL; *n* = 16) compared to patients with body weight stable (BWS; *n* = 12) (*p* = 0.031) and vs. controls (*n* = 10) (*p* < 0.001). **(C)** Combined analysis of patients presenting both BWL and AN (n = 11) showed significantly higher circulating LCN2 levels compared to those with BWS without AN (*n* = 9) (*p* = 0.046). LCN2, lipocalin 2; w/o AN, without anorexia; AN, anorexia; BWL, body weight loss; BWS, body weight stable.

**Table 2:** Spearman’s correlations analysis of lipocalin 2 with cytokines in gastrointestinal cancer patients according to anorexia status assessed by the Functional Assessment of Anorexia/Cachexia Therapy (FAACT) questionnaire and to self-reported 6-month weight changes prior to the enrollment.

	LCN2 (ng/mL) AN (*n* = 14)	LCN2 (ng/mL) w/o AN (*n* = 14)	LCN2 (ng/mL) BWL (*n* = 16)	LCN2 (ng/mL) BWS (*n* = 12)
IL-6 (pg/mL)	*rho* = 0.837 *p* < 0.001	*rho* = 0.873 *p* < 0.001	*rho* = 0.915 *p* < 0.001	*rho* = 0.846 *p* < 0.001
IL-8 (pg/mL)	*rho* = 0.846 *p* < 0.001	*rho* = 0.631 *p* = 0.016	*rho* = 0.797 *p* < 0.001	*rho* = 0.720 *p* = 0.008
IL-10 (pg/mL)	*rho* = − 0.785 *p* < 0.001	*rho* = − 0.855 *p* < 0.001	*rho* = − 0.797 *p* < 0.001	*rho* = − 0.916 *p* < 0.001
IL-15 (pg/mL)	*rho* = 0.612 *p* = 0.020	*rho* = 0.640 *p* = 0.014	*rho* = 0.512 *p* = 0.043	*rho* = 0.650 *p* = 0.022
IL-18 (pg/mL)	*rho* = −0.486 *p* = 0.078	*rho* = −0.618 *p* = 0.019	*rho* = −0.491 *p* = 0.053	*rho* = −0.594 *p* = 0.042
TNF- α (pg/mL)	*rho* = 0.306 *p* = 0.288	*rho* = 0.688 *p* = 0.007	*rho* = 0.616 *p* = 0.011	*rho* = 0.587 *p* = 0.045

LCN2, lipocalin 2; AN, anorexia; w/o AN, without anorexia; BWL, body weight loss; BWS, body weight stable; IL-6, interleukin-6; IL-8, interleukin-8; IL-10, interleukin-10; IL-15, interleukin-15; IL-18, interleukin-18; TNF-α, tumor necrosis factor alpha.

Given that involuntary weight loss represents a hallmark of cachexia, cancer patients were stratified according to self-reported 6-month weight change prior to the enrollment. Serum LCN2 levels were significantly higher in patients with body weight loss compared with weight-stable patients (*p* = 0.031) and with respect to controls (*p* < 0.001) (Figure 3B). This stratification further revealed significant correlations between circulating LCN2 levels and inflammatory cytokines (Table 2 - right side).

When combining the feature of anorexia with body weight loss, LCN2 levels were found higher compared to the feature of no anorexia and body weight stable (*p* = 0.046) (Figure 3C).

### Circulating lipocalin 2 in association with the level of muscularity and body weight change

Gastrointestinal cancer patients were stratified according to skeletal muscle mass as presenting with low or high muscle mass based on median value of L3-SMI (internal sex-specific cut-off was set at 47.89 cm^2^/m^2^ for males and at 39.14 cm^2^/m^2^ for females).

A total of 25/28 patients were included in the body-composition analysis; three patients were excluded because CT-scan data were unavailable.

Our results showed that serum LCN2 levels were significantly higher in gastrointestinal cancer patients with low muscle mass compared with controls (*p* = 0.007) (Figure 4A).

**Figure 4:**
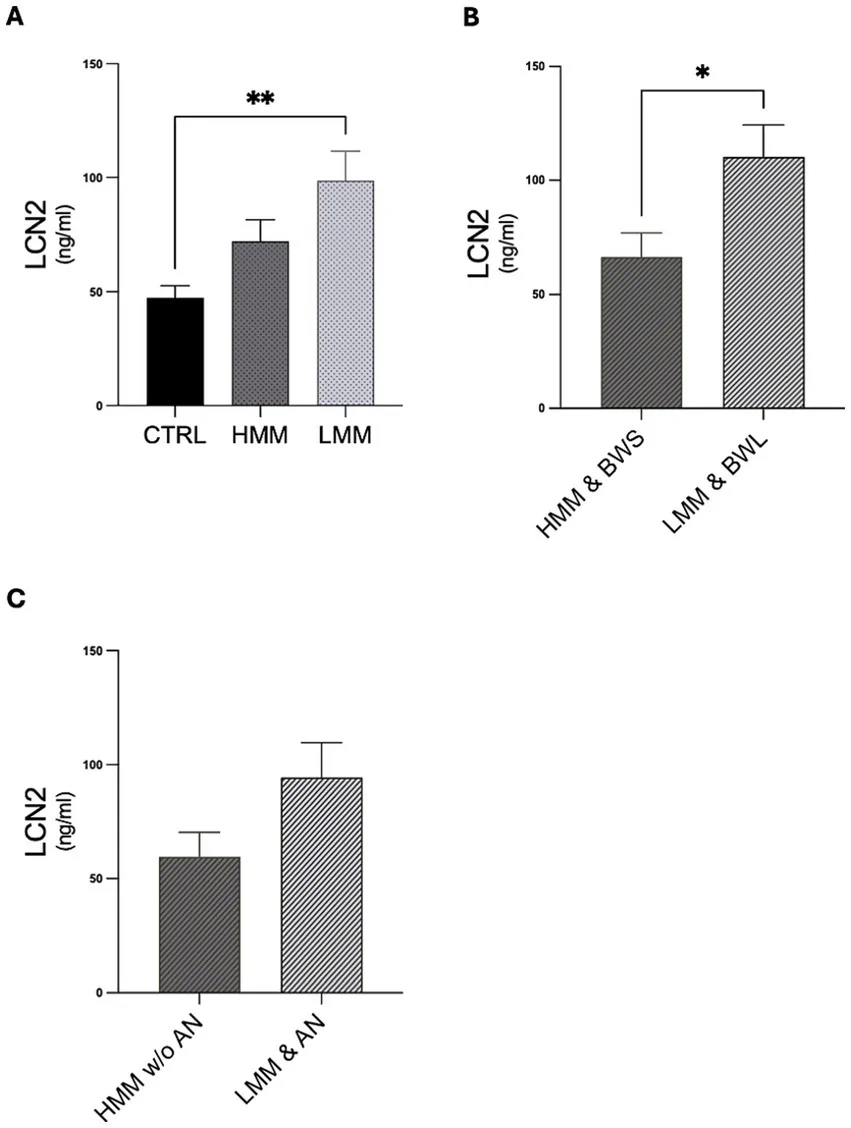
Circulating lipocalin 2 levels in association with skeletal muscle mass and nutritional and clinical phenotypes in gastrointestinal cancer patients and controls. **(A)** Circulating LCN2 levels were analyzed in gastrointestinal cancer patients stratified according to skeletal muscle mass assessed by CT using sex-specific L3-SMI cut-offs (median value of internal sex-specific cut-off: 47.89 cm^2^/m^2^ for males and 39.14 cm^2^/m^2^ for females). Patients were classified as having low muscle mass (LMM; *n* = 12), high muscle mass (HMM; *n* = 13) compared to controls (*n* = 10). Data shown significant higher LCN2 levels in LMM vs. controls (*p* = 0.007). **(B)** Data shown increased levels of LCN2 in patients with combined LMM and BWL (*n* = 9) vs. those with HMM and BWS (*n* = 8) (*p* = 0.028). **(C)** No significant differences were observed for patients stratified according to combined LMM and AN (*n* = 8) vs. those with HMM and w/o AN (*n* = 8). LCN2, lipocalin 2; CT, computed tomography; L3-SMI, skeletal muscle index at the third lumbar vertebra; LMM, low muscle mass; HMM, high muscle mass; BWL, body weight loss; BWS, body weight stable; AN, anorexia; w/o AN, without anorexia.

To further explore the implication of LCN2 in nutritional impairment in our cohort, we analyzed the LCN2 circulating levels relation with combined level of muscularity and body weight change.

The LCN2 levels resulted significantly higher in patients presenting combined phenotypes of low muscle mass with body weight loss compared with weight stable and higher muscularity levels (*p* = 0.028) (Figure 4B).

In contrast, no significant differences in circulating LCN2 levels were observed between patients with combined low muscle mass and anorexia and those with high muscle mass without anorexia (Figure 4C). Finally, in multivariable logistic regression analysis (Table 3), circulating LCN2 remained significantly associated with body weight loss after adjustment for age, sex, and CRP (OR = 1.043; 95% CI 1.006–1.082; *p* = 0.023). Age, sex, and CRP were not significantly associated with body weight loss in the same model.

**Table 3:** Multivariable binary logistic regression for factors associated with body weight loss in gastrointestinal cancer patients (*n* = 28).

Variable	OR	95% CI	*p*-value
Age (years)	1.063	0.978–1.155	0.149
CRP (mg/dL)	1.077	0.932–1.245	0.316
Sex (female vs. male)	0.098	0.008–1.225	0.071
LCN2 (ng/mL)	1.043	1.006–1.082	0.023

OR, odds ratio; CI, confidence interval; LCN2, lipocalin-2. Odds ratios are per one-unit increase for continuous predictors. Sex is coded with male as reference.

## Discussion

In the present study, circulating LCN2 levels were found elevated in patients with gastrointestinal cancer compared with non-cancer controls and were closely associated with systemic inflammation, anorexia, nutritional impairment, and with lower muscularity. In this light, our findings, predominantly obtained in colorectal cancer, suggest a potential role for LCN2 as a circulating marker of cancer-associated cachexia severity and reinforce its relevance within the inflammatory-nutritional axis characterizing this syndrome. These findings are in line with previous data indicating LCN2 as a candidate biomarker in pancreatic cancer and cachexia-related clinical deterioration (9, 13).

A key finding of our work is the robust increase in serum LCN2 levels in gastrointestinal cancer patients, accompanied by a pronounced pro-inflammatory cytokine profile. Elevated circulating levels of IL-6, IL-8, IL-15, and TNF-α observed in cancer patients are consistent with the established inflammatory landscape of cancer cachexia (14, 15). Importantly, our analyses revealed strong and consistent positive correlations between LCN2 and these pro-inflammatory cytokines across the cancer cohort, suggesting that circulating LCN2 may closely mirror the magnitude of systemic inflammatory activation rather than isolated cytokine changes.

Among the cytokines analyzed, IL-6 showed the strongest correlation with circulating LCN2 levels. IL-6 is a key driver of the hepatic acute-phase response, energy expenditure dysregulation, and muscle protein catabolism, and has been repeatedly implicated in the pathogenesis of cancer cachexia (7, 16). The tight association between IL-6 and LCN2 observed in our cohort suggests that LCN2 may reflect IL-6 driven inflammatory signaling, positioning LCN2 downstream or in parallel with canonical cachexia-related inflammatory pathways.

Similarly, the strong correlation between LCN2 and IL-8 is particularly noteworthy. IL-8 plays a central role in neutrophil recruitment, tumor-associated inflammation, and angiogenesis (17), and elevated IL-8 levels have been linked to disease severity and poor prognosis in gastrointestinal malignancies (18). Given that LCN2 is abundantly expressed by neutrophils and released during neutrophil activation (7), the close association between IL-8 and LCN2 supports a translational link between neutrophil-driven inflammatory response and systemic metabolic derangements characteristic of cancer cachexia. This is also supported by clinical data showing positive correlations between LCN2 and neutrophil activation markers, including calprotectin, myeloperoxidase, elastase, and bactericidal/permeability-increasing protein, in patients with pancreatic ductal adenocarcinoma (13).

LCN2 also correlated positively with IL-15 and TNF-α, cytokines known to exert pleiotropic and, in part, opposing effects on immune activation and tissue metabolism. TNF-α is a well-established mediator of muscle proteolysis and anorexia in cancer cachexia (19). In contrast, IL-15 has been shown in experimental models to promote myogenesis and to counteract TNF-α-induced catabolic signaling in cultured myotubes (20), suggesting a potential protective role in muscle homeostasis under inflammatory conditions. The concurrent elevation of IL-15 and TNF-α, together with their positive correlation with circulating LCN2 in our cohort may therefore reflect a dysregulated inflammatory environment in which compensatory, muscle-preserving signals coexist with dominant catabolic pathways but likely fail to restore metabolic balance. Along this line, IL-15 upregulation may represent an insufficient or ineffective adaptive response to ongoing inflammation-driven tissue wasting rather than a marker of preserved muscle integrity.

In contrast, LCN2 levels were inversely correlated with IL-10 and IL-18, highlighting a potential imbalance between pro-inflammatory and regulatory immune signals. IL-10, a key anti-inflammatory cytokine, is involved in limiting immune-mediated tissue damage (21), and its inverse association with LCN2 suggests that higher LCN2 levels may identify patients with insufficient counter-regulatory inflammatory control. Similarly, the pro-inflammatory cytokine IL-18, while context-dependent, has been implicated in immune regulation and metabolic homeostasis (22, 23), and its negative association with LCN2 may further reflect a shift toward sustained inflammatory activation. Together, these opposing correlations indicate that LCN2 may act as a surrogate marker of inflammatory dominance rather than generalized immune activation.

Beyond inflammation, our data highlight a potential association between circulating LCN2 levels and the main clinical features of cancer cachexia such as poor appetite and involuntary weight loss (24), suggesting that LCN2 may reflect the convergence of inflammatory and metabolic dysregulation underlying negative energy balance.

In our cohort, patients experiencing involuntary body weight loss exhibited significantly higher LCN2 levels compared with weight-stable patients, indicating that LCN2 elevation may accompany early nutritional deterioration rather than isolated inflammatory activation.

The association between circulating LCN2 and body weight loss remained significant after adjustment for age, sex, and CRP in multivariable analysis. This finding suggests that the relationship between LCN2 and weight loss may not be solely attributable to differences in age or to the degree of systemic inflammation reflected by CRP. Notably, within weight-loss subgroups, LCN2 remained significantly associated with pro-inflammatory cytokines, suggesting that inflammation-linked LCN2 upregulation persists across different stages of weight loss and may contribute to progressive energy imbalance.

In our cohort, anorexia was associated with significantly higher circulating LCN2 levels compared with controls, but not with non-anorexic patients, and subgroup analyses revealed sustained correlations between LCN2 and inflammatory cytokines. These results align with experimental data suggesting that LCN2 influences central appetite regulation (25), supporting its translational relevance as a marker associated with anorectic phenotype in cancer patients. This is consistent with previous work implicating LCN2 in appetite regulation and energy metabolism across cachexia-related conditions (26).

Importantly, the highest LCN2 levels were observed in patients presenting a combined phenotype of anorexia and body weight loss, the most severe cachectic state associated with profound metabolic dysregulation and poor prognosis (3).

Skeletal muscle wasting is a defining component of cancer cachexia and a major determinant of functional decline and survival (27). In this context, our observation that LCN2 levels were significantly elevated in patients with low skeletal muscle mass further strengthens the link between LCN2 and tissue catabolism. Given the strong correlations between LCN2 and pro-inflammatory cytokines, it is plausible that elevated LCN2 reflects an inflammation-driven catabolic environment conducive to muscle wasting. Notably, LCN2 levels were particularly increased in patients presenting combined phenotypes of low muscle mass with body weight loss or high nutritional risk, conditions that reflect advanced metabolic impairment. These findings are consistent with previous reports suggesting a role for LCN2 in muscle atrophy and support the hypothesis that circulating LCN2 may reflect ongoing muscle wasting in cancer patients.

Surprisingly, no significant differences in LCN2 levels were observed between patients with combined low muscle mass and anorexia compared with those with preserved muscle mass and no anorexia. This finding may suggest that the relationship between LCN2 and cachexia-related phenotypes may be more strongly driven by objective metabolic and nutritional deterioration, such as weight loss and nutritional risk, rather than by anorexia alone. This distinction may be clinically relevant, as it highlights the multifaceted nature of cachexia and the need to consider integrated phenotypes when evaluating potential biomarkers. In addition, by excluding patients with impaired renal function, we ruled out the potential confounding role of renal functionality in the interpretation of higher LCN2 circulating levels (28, 29).

Several limitations of this study should be acknowledged. The relatively small sample size and the cross-sectional design limit causal inference and preclude assessment of temporal relationships between LCN2 elevation and cachexia progression. Particularly, the subgroup stratification and the predominance of colorectal cancer limit the generalizability of the present findings. Although not statistically significant, the numerical difference in median age between cancer patients and controls may still represent a potential source of residual confounding. The skeletal muscle mass was assessed using sex-specific internal cut-offs rather than established external reference values, which may affect comparability with other cohorts. Moreover, the observational nature of the study does not allow mechanistic conclusions regarding the causal role of LCN2 in anorexia or muscle wasting, underscoring the need for dedicated investigations. Finally, functional outcomes and survival were not evaluated and warrant investigation in future longitudinal studies. Despite these limitations, our study provides novel clinical evidence linking circulating LCN2 levels to systemic inflammation, anorexia, nutritional risk, and skeletal muscle mass in gastrointestinal cancer patients.

## Conclusion

Our study provides novel translational evidence that circulating LCN2 is tightly linked to key inflammatory pathways and to clinical manifestations of cachexia in gastrointestinal cancer patients, particularly in colorectal presentation. These findings suggest that LCN2 may represent a promising biomarker integrating inflammatory burden and metabolic deterioration. Further longitudinal and mechanistic studies are warranted to determine whether LCN2 actively contributes to cachexia pathophysiology or primarily reflects inflammatory stress, and to evaluate its potential utility in patient stratification and therapeutic monitoring.

## Data Availability

The raw data supporting the conclusions of this article will be made available by the authors, without undue reservation.
