# Supplementary material for: Circulating lipocalin-2 is associated with systemic inflammation and cachexia-related features in patients with gastrointestinal cancer
Source: Front Nutr. 2026 Oct 19;13:1937525. doi: 10.3389/fnut.2026.1937525 (PMC13724323; doi:10.3389/fnut.2026.1937525)
Supplement: Supplementary file 1 [file Table_1.DOCX]

## Supplementary Table 1. Characteristics of anorexic vs non-anorexic patients

|  | **Anorexic (n=14)** | **Non-anorexic (n=14)** | **p-value** |
| --- | --- | --- | --- |
| Male, n (%) | 11 (78.6) | 8 (57.1) | *p = 0.225* |
| Age (years), median (Q1; Q3) | 75.0 (56.8; 85.0) | 69.5 (51.5; 78.3) | *p = 0.210* |
| Actual weight, kg, median (Q1; Q3) | 68.0 (64.0; 73.8) | 79.0 (61.3; 82.0) | *p = 0.325* |
| BMI (kg/m²), mean ± SD | 22.8 ± 2.7 | 25.5 ± 4.6 | *p = 0.095* |
| **Blood parameters, median (Q1; Q3)** |  |  |  |
| C-reactive protein, mg/dL | 5.08 (2.66; 15.99) | 3.91 (0.19; 12.48) | *p = 0.603* |
| Hemoglobin, g/dL | 11.95 (10.35; 12.80) | 13.55 (10.38; 14.63) | *p = 0.114* |
| Albumin, g/dL | 3.30 (2.75; 3.55) | 3.60 (3.48; 4.00) | *p = 0.019* |
| Creatinine, mg/dL | 0.96 (0.79; 1.31) | 0.80 (0.64; 1.39) | *p = 0.482* |
| **Main comorbidities, n (%)** |  |  |  |
| Diabetes mellitus | 2 (14.3) | 3 (21.4) | *p = 0.622* |
| Dyslipidemia | 3 (21.4) | 3 (21.4) | *p > 0.99* |
| **Type of cancer, n (%)** |  |  | *p = 0.513* |
| Colorectal | 12 (85.7) | 12 (85.7) |  |
| Gastric | 1 (7.1) | 2 (14.3) |  |
| Pancreas | 1 (7.1) | 0 (0.0) |  |
| **Stage of the disease, n (%)** |  |  | *p = 0.127* |
| I–II | 6 (42.8) | 10 (71.4) |  |
| III–IV | 8 (57.1) | 4 (28.6) |  |

## Supplementary Table 2. Characteristics of patients with cachexia vs without cachexia

|  | **Cachexia (n=16)** | **Weight stable (n=12)** | **p-value** |
| --- | --- | --- | --- |
| Male, n (%) | 13 (81.3) | 6 (50.0) | *p = 0.080* |
| Age (years), median (Q1; Q3) | 76.0 (65.0; 85.3) | 55.5 (48.5; 78.0) | *p = 0.037* |
| Actual weight, kg, median (Q1; Q3) | 72.5 (64.0; 81.3) | 67.5 (61.5; 81.5) | *p = 0.719* |
| BMI (kg/m²), mean ± SD | 24.8 ± 4.3 | 23.7 ± 3.7 | *p = 0.705* |
| **Blood parameters, median (Q1; Q3)** |  |  |  |
| C-reactive protein, mg/dL | 5.60 (2.48; 13.85) | 3.91 (0.16; 12.32) | *p = 0.397* |
| Hemoglobin, g/dL | 11.45 (10.10; 12.98) | 13.95 (11.38; 14.68) | *p = 0.017* |
| Albumin, g/dL | 3.30 (2.78; 3.50) | 3.70 (3.45; 4.00) | *p = 0.004* |
| Creatinine, mg/dL | 0.98 (0.77; 1.54) | 0.80 (0.59; 1.01) | *p = 0.146* |
| **Main comorbidities, n (%)** |  |  |  |
| Diabetes mellitus | 4 (25.0) | 1 (8.3) | *p = 0.254* |
| Dyslipidemia | 5 (31.3) | 1 (8.3) | *p = 0.144* |
| **Type of cancer, n (%)** |  |  | *p = 0.482* |
| Colorectal | 14 (87.5) | 10 (83.3) |  |
| Gastric | 1 (6.3) | 2 (16.7) |  |
| Pancreas | 1 (6.3) | 0 (0.0) |  |
| **Stage of the disease, n (%)** |  |  | *p = 0.098* |
| I–II | 7 (43.8) | 9 (75.0) |  |
| III–IV | 9 (56.3) | 3 (25.0) |  |

## Supplementary Table 3. Characteristics of patients with low vs high muscle mass

|  | **Low muscle mass (n=12)** | **High muscle mass (n=13)** | **p-value** |
| --- | --- | --- | --- |
| Male, n (%) | 9 (75.0) | 9 (69.2) | *p = 0.748* |
| Age (years), median (Q1; Q3) | 67.0 (53.0; 79.0) | 73.0 (54.0; 81.0) | *p = 0.936* |
| Actual weight, kg, median (Q1; Q3) | 71.0 (63.0; 81.0) | 67.0 (62.0; 82.0) | *p = 0.459* |
| BMI (kg/m²), mean ± SD | 24.3 ± 2.8 | 23.4 ± 4.0 | *p = 0.459* |
| **Blood parameters, median (Q1; Q3)** |  |  |  |
| C-reactive protein, mg/dL | 11.37 (3.35; 12.70) | 2.78 (0.19; 10.49) | *p = 0.186* |
| Hemoglobin, g/dL | 12.80 (10.50; 13.60) | 11.90 (10.15; 13.95) | *p = 0.728* |
| Albumin, g/dL | 3.50 (2.70; 4.00) | 3.60 (3.35; 3.81) | *p = 0.406* |
| Creatinine, mg/dL | 0.85 (0.72; 1.88) | 0.84 (0.61; 1.17) | *p = 0.320* |
| **Main comorbidities, n (%)** |  |  |  |
| Diabetes mellitus | 2 (16.7) | 2 (15.4) | *p = 0.930* |
| Dyslipidemia | 3 (25.0) | 2 (15.4) | *p = 0.548* |
| **Type of cancer, n (%)** |  |  | *p = 0.564* |
| Colorectal | 10 (83.3) | 12 (92.3) |  |
| Gastric | 1 (8.3) | 1 (7.7) |  |
| Pancreas | 1 (8.3) | 0 (0.0) |  |
| **Stage of the disease, n (%)** |  |  | *p = 0.327* |
| I–II | 6 (50.0) | 9 (69.2) |  |
| III–IV | 6 (50.0) | 4 (30.8) |  |
